# Proto-Adapter: Efficient Training-Free CLIP-Adapter for Few-Shot Image Classification

**DOI:** 10.3390/s24113624

**Published:** 2024-06-04

**Authors:** Naoki Kato, Yoshiki Nota, Yoshimitsu Aoki

**Affiliations:** 1 Department of Electrical Engineering, Faculty of Science and Technology, Keio University, 3-14-1 Hiyoshi, Kohoku-ku, Yokohama 223-8522, Kanagawa, Japan; aoki@elec.keio.ac.jp; 2 Meidensha Corporation, Tokyo 141-6029, Japan; nota-y@mb.meidensha.co.jp

**Keywords:** few-shot learning, image classification, foundation models

## Abstract

Large vision-language models, such as Contrastive Vision-Language Pre-training (CLIP), pre-trained on large-scale image–text datasets, have demonstrated robust zero-shot transfer capabilities across various downstream tasks. To further enhance the few-shot recognition performance of CLIP, Tip-Adapter augments the CLIP model with an adapter that incorporates a key-value cache model constructed from the few-shot training set. This approach enables training-free adaptation and has shown significant improvements in few-shot recognition, especially with additional fine-tuning. However, the size of the adapter increases in proportion to the number of training samples, making it difficult to deploy in practical applications. In this paper, we propose a novel CLIP adaptation method, named Proto-Adapter, which employs a single-layer adapter of constant size regardless of the amount of training data and even outperforms Tip-Adapter. Proto-Adapter constructs the adapter’s weights based on prototype representations for each class. By aggregating the features of the training samples, it successfully reduces the size of the adapter without compromising performance. Moreover, the performance of the model can be further enhanced by fine-tuning the adapter’s weights using a distance margin penalty, which imposes additional inter-class discrepancy to the output logits. We posit that this training scheme allows us to obtain a model with a discriminative decision boundary even when trained with a limited amount of data. We demonstrate the effectiveness of the proposed method through extensive experiments of few-shot classification on diverse datasets.

## 1. Introduction

Few-shot image classification aims to classify unseen images using models trained with a limited number of labeled examples. This task is crucial in fields where collecting extensive data is challenging, such as medical image diagnosis and visual inspection. Addressing few-shot image classification typically involves data augmentation [1,2], transfer learning [3,4,5], and meta-learning [6,7]. Among these, meta-learning is a fundamental research area developed to enhance few-shot learning efficiency. It focuses on creating a data-efficient learner from the source (meta-train) dataset by simulating few-shot learning scenarios and then applying this specialized learner to the target (meta-test) set. Many methods [8] utilize meta-train sets that differ from the target set in classes but are domain-similar, offering a larger sample size than the few-shot training data. For example, the meta-train/test set of miniImageNet [9] comprises different ImageNet [10] classes, and CIFAR-FS [11] is formed by dividing CIFAR-100 [12]. Using a meta-train set enhances the model’s performance by providing domain-specific knowledge not sufficiently covered by few-shot training data alone. However, real-world data availability is often limited, necessitating few-shot learning methods that rely solely on the few-shot training set of the target classes. Therefore, there’s a need for a few-shot learning method that relies solely on the few-shot training set of the target classes as the task-related data.

Conversely, Contrastive Vision-Language Pre-training (CLIP) [13], a vision-language pre-trained model renowned for its strong transferability, has gained prominence in the field of computer vision. It enables zero-shot inference on data across arbitrary categories through contrastive pre-training on extensive image–text pairs. It is shown that linear probing with few-shot data can improve classification performance on downstream tasks. The few-shot transferability of CLIP is particularly advantageous in practical applications, as it obviates the need for additional target task data, such as a meta-train set. Recent years have seen several studies aimed at further improving the CLIP few-shot recognition capabilities. CLIP-Adapter [14] adjusts CLIP for downstream tasks by incorporating a residual-style adapter [15] atop the image and text encoders. This approach involves freezing the weights of the pre-trained encoders and training the adapter’s weights using few-shot data from downstream tasks. Context Optimization (CoOp) [16] enhances the text encoder’s manually designed input prompts in CLIP through optimization with few-shot data. Meanwhile, Tip-Adapter [17] enables CLIP adaptation to downstream tasks without stochastic gradient descent (SGD) by constructing additional layers with a key-value cache model. If further fine-tuning is feasible, adjusting the adapter’s weights can significantly enhance few-shot classification performance. However, the adapter’s size scales with the number of training samples, posing challenges for real-world deployment.

In this paper, we propose Proto-Adapter, a novel adaptation method for CLIP that inherits the training-free property of Tip-Adapter while overcoming its challenges. Proto-Adapter augments CLIP with an adapter consisting of a single linear layer. It initializes the adapter using prototype vectors that aggregate the features of each class in the few-shot training data. This approach ensures that the adapter’s size remains constant relative to the number of training samples.

**Figure 1:**
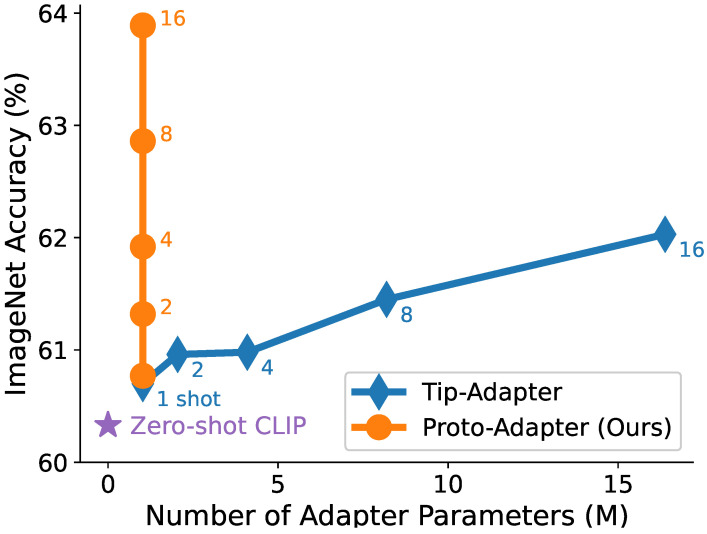
Parameters versus accuracy on ImageNet varying numbers of training samples per class. Both methods can adapt the CLIP [13] model to downstream tasks without gradient descent training. Our Proto-Adapter consistently outperforms Tip-Adapter [17], despite having a constant, small number of adapter parameters, regardless of the amount of training data.

Figure 1 illustrates the performance comparison between Tip-Adapter and Proto-Adapter in different few-shot settings. Remarkably, despite its simplicity and lightweight structure, Proto-Adapter surpasses the performance of Tip-Adapter. We also propose fine-tuning the adapter weights with the Additive Angular Margin Penalty [18], a deep metric learning method often used in face recognition, to further enhance few-shot performance. By introducing this penalty, we expect to obtain a model with a discriminative decision boundary, even when trained with a small amount of data. We conduct extensive experiments of few-shot classification on ImageNet and 10 other datasets to demonstrate the effectiveness of the proposed method.

In summary, the contributions of our work are as follows:

- We introduce a novel training-free adaptation method for CLIP that maintains a constant adapter size irrespective of the number of training samples. This is achieved by aggregating the features of training samples within each class to construct the adapter weights.
- The proposed method’s performance can be further enhanced by fine-tuning it with Additive Angular Margin Penalty, which introduces additional inter-class discrepancy to the output logits.
- We evaluate our method on ImageNet and 10 additional datasets for few-shot classification, demonstrating its superiority over existing CLIP adaptation methods.

## 2. Methods

In this section, we first briefly revisit Tip-Adapter. Then, we specifically introduce the proposed method.

### 2.1. Revisiting Tip-Adapter

Tip-Adapter [17] is a training-free and non-parametric extension of CLIP-Adapter [14]. Following CLIP-Adapter, it appends a lightweight two-layer Multi-layer Perceptron (MLP) to the pre-trained fixed-weight CLIP model [13] and predicts the adapted feature residuals for each input image. Additionally, Tip-Adapter constructs a key-value cache model from the few-shot training set and transforms the cache into the weights of the adapter MLP in a non-parametric manner without SGD training. In addition, if fine-tuning is allowable, further fine-tuning with such weights as network initialization is able to achieve much higher performance.

Given the pre-trained CLIP model and a *N*-class *K*-shot training set for few-shot classification, there are *K* annotated images in each of the *N* categories, denoted as $I_{N,K}$ with their labels $L_{N,K}$. Tip-Adapter constructs a key-value cache model and converts it to obtain the weights of the adapter layers. Specifically, for all NK training samples, it utilizes CLIP to extract *D*-dimensional L2 normalized visual features of the few-shot training images to create keys $F_{\mathrm{train}}\in R^{D\times NK}$ as

(1) $$F_{\mathrm{train}}={\mathrm{CLIP}}_{vis}\left(I_{N,K}\right),$$

where ${\mathrm{CLIP}}_{vis}$ denotes the CLIP image encoder. Values $L_{\mathrm{train}}\in R^{N\times NK}$ are obtained by converting the labels $L_{N,K}$ of the few-shot training data into one-hot encodings.

After constructing the cache model, the adapter weights are determined according to cached key-value pairs. Hence, the adapter’s logits for a query image can be estimated as

(2) $$p_{\mathrm{train}}=L_{\mathrm{train}}\varphi \left(F_{\mathrm{train}}^{T}f\right),$$

where φ(x)=exp(−β(1−x)) with a modulating hyper-parameter β denotes the activation function in the MLP, and $f\in R^{D}$ is the L2 normalized visual feature of the query image extracted by the CLIP image encoder.

The final prediction is then computed by a linear combination of the adapter’s logits $p_{\mathrm{train}}$ and the CLIP zero-shot logits as

(3) $$\mathrm{logits}=\alpha p_{\mathrm{train}}+W_{\mathrm{text}}^{T}f,$$

where α is the mixing coefficient and $W_{\mathrm{text}}\in R^{D\times N}$ denotes the weights of the CLIP text classifier. Following zero-shot CLIP, $W_{\mathrm{text}}$ is constructed by placing each category name into the pre-defined prompt template and encodes them by the CLIP pre-trained textual encoder.

### 2.2. Proposed Method

**Figure 2:**
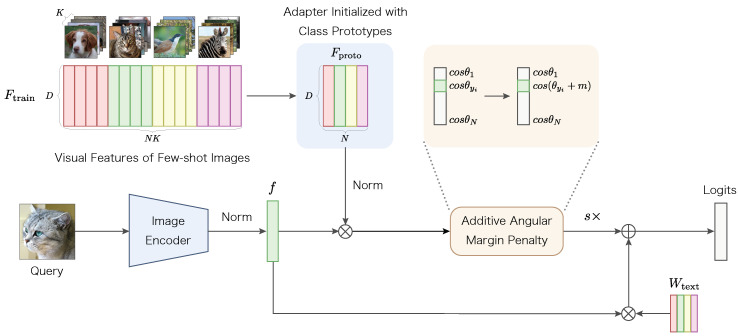
Overview of the proposed Proto-Adapter. Visual features from few-shot training images are aggregated to construct a prototype vector for each class. These vectors serve as the weights for an adapter layer placed on top of the CLIP image encoder. Should further fine-tuning be possible, we suggest fine-tuning the adapter layer with an Additive Angular Margin Penalty [18]. This approach aims to achieve more discriminative predictions, even with a limited amount of training data.

We propose Proto-Adapter, which retains the training-free property of Tip-Adapter [17] and has a more simple and lightweight architecture adapter. The overall pipeline of Proto-Adapter is shown in Figure 2. By using the prototype representation of each class to construct adapter weights, the size of the adapter is small and invariant to the number of training samples. In addition, fine-tuning with the Additive Angular Margin Penalty can further improve the recognition performance in the few-shot training setting. We describe the details of the proposed method below.

#### 2.2.1. Prototype-Based Adapter

Proto-Adapter employs only a single-layer adapter on the top of the CLIP image encoder. The adapter weights $F_{\mathrm{proto}}\in R^{D\times N}$ are created from the concatenation of the *D*-dimensional prototype representation $c_{n}\in R^{D}$ of each class n∈{1,⋯,N} as

(4) $$\begin{array}{c} F_{\mathrm{proto}}=\left[c_{1},c_{2},\;\cdots ,c_{N}\right]. \end{array}$$

Each prototype is the mean vector of the visual features of training images belonging to its class extracted by the CLIP image encoder:

(5) $$\begin{array}{c} c_{n}=\frac{1}{K}\sum_{k}^{K}{\mathrm{CLIP}}_{vis}\left(I_{n,k}\right), \end{array}$$

where $I_{n,k}$ is a training image of the class *n*. We find that proper normalization of the adapter weights significantly improves the performance of the model, which we experiment with in Section 3.4.

Given a query image with visual features *f*, the logits of the adapter are simply calculated by the matrix product of the visual features and the adapter weights:

(6) $$p_{\mathrm{proto}}=F_{\mathrm{proto}}^{T}f.$$

Finally, the prediction logits are obtained by a linear combination of the adapter’s logits and the CLIP logits as

(7) $$\mathrm{logits}=\alpha p_{\mathrm{proto}}+W_{\mathrm{text}}^{T}f.$$

**Figure 3:**
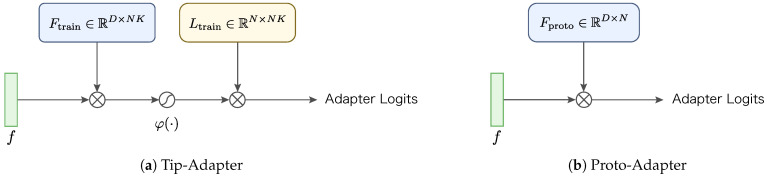
Comparison of the architectures of Tip-Adapter and Proto-Adapter. (**a**) Tip-Adapter consists of an adapter with two linear layers and a single activation function, with the size of each linear layer increasing in proportion to the number of training data. (**b**) In contrast, Proto-Adapter is composed solely of a single fixed-size linear layer adapter.

We show the architecture comparison of Tip-Adapter and Proto-Adapter in Figure 3. By aggregating image features for each class in the training data into a prototype representation, our proposed Proto-Adapter can infer test data without SGD training. It employs a single-layer adapter that operates without activation functions or additional hyper-parameters.

#### 2.2.2. Fine-Tuning with Additive Angular Margin Penalty

Like the Tip-Adapter, the proposed method can enhance recognition performance by fine-tuning the adapter weights with few-shot training data. However, we are concerned that the decision boundaries obtained from training with a limited number of examples may not be robust enough to classify a diverse set of test data accurately. To address this issue, we introduce a fine-tuning approach that achieves a highly discriminative model, even with limited data, by employing a metric learning technique commonly utilized in face recognition. Specifically, we fine-tune the adapter weights $W=F_{\mathrm{proto}}$ using the Additive Angular Margin Penalty proposed in ArcFace [18].

Given the normalized visual features $f_{i}$ of the *i*-th sample belonging to the $y_{i}$-th class, the logit for the *j*-th class can be represented as $W_{j}^{T}f_{i}=\cos\theta_{j}$, where $W_{j}\in R^{D}$ denotes the *j*-th column of the adapter weights *W*, and $\theta_{j}$ is the angle between the L2 normalized weight $W_{j}$ and the feature $f_{i}$. Following ArcFace, we add an Additive Angular Margin Penalty *m* between $f_{i}$ and $W_{y_{i}}$ to obtain the logit for the positive class $y_{i}$ as

(8) $${\mathrm{logit}}_{y_{i}}=\cos\left(\theta_{y_{i}}+m\right).$$

We then apply the cross-entropy loss to the obtained logits to fine-tune the adapter weights *W*. The application of the Additive Angular Margin Penalty modifies the adapter weights to enhance the separation between positive and negative examples. It is important to note that the Additive Angular Margin Penalty is applied only during training; thus, this approach does not alter the inference pipeline or increase the computational cost for inference.

## 3. Results

In this section, we present a comprehensive evaluation of Proto-Adapter through experiments across multiple benchmarks and report the findings.

### 3.1. Experimental Settings

**Datasets.** We evaluate the proposed method on 11 publicly available image classification datasets used in CLIP [13]: ImageNet [10], StanfordCars [19], UCF101 [20], Caltech101 [21], Flowers102 [22], SUN397 [23], DTD [24], EuroSAT [25], FGVCAircraft [26], OxfordPets [27], and Food101 [28]. These datasets encompass a variety of visual tasks, from classifying general objects, scenes, and actions to more detailed categories, along with specific tasks such as identifying textures and satellite images.

**Implementation details.** Like Tip-Adapter, the proposed Proto-Adapter is available in two versions: one is training-free, and the other includes additional fine-tuning. Each version is implemented according to the method described in Section 2.2. In this section, we refer to these versions as Proto-Adapter and Proto-Adapter-F, respectively. Following the few-shot evaluation protocol used in CLIP, we train our model with 1, 2, 4, 8, and 16 shots and test it on the full test sets. For the CLIP backbone, we employ ResNet-50 [29] as the image encoder and a transformer [30] as the textual encoder, consistent with the comparison methods. We use prompt ensembling [13] to create prompts for CLIP, which involves inputting multiple templates into the CLIP textual encoder and then averaging the text features. The templates for each dataset are the same as those used for Tip-Adapter. For fine-tuning the adapter weights with Additive Angular Margin Loss, we train Proto-Adapter using the Adam optimizer [31] with a batch size of 256 for 20 epochs. The learning rate starts from 4 × 10^−4^ and decreases to 4 × 10^−5^ following a cosine annealing learning rate schedule. We tune other hyperparameters, such as angular margin penalty *m* and α, for each dataset using the validation set. Consistent with Tip-Adapter, our data augmentation strategy includes random cropping, resizing, and random horizontal flipping.

### 3.2. Comparison on ImageNet

We first compare Proto-Adapter with other CLIP-based adaptation methods on ImageNet [10], one of the representative datasets for image classification. Comparison methods are Zero-shot CLIP [13], Linear-probe CLIP [13], CoOp [16], CLIP-Adapter [14], and Tip-Adapter [17]. Zero-shot CLIP does not need extra training on downstream datasets. Instead, it performs zero-shot classification based on the similarities between the image features of test images and the text features of the hand-crafted prompts. Linear-probe CLIP freezes the original image encoder and trains only a logistic regression classifier attached to it using few-shot training data. To automate prompt engineering, CoOp learns context words in text prompts. We use the best-performing variant, named the unified context version, which shares the same context across all classes. CLIP-Adapter features a simple architectural design, consisting of residual-style feature adapters added on top of the frozen image–text encoder. Tip-Adapter has an architecture similar to CLIP-Adapter, but it appends an adapter only to the image branch and initializes the adapter layers using the image features and labels from the few-shot training set. All these methods are based on the pre-trained CLIP [13] with ResNet-50 [29] as image encoders. Like Proto-Adapter, prompt ensembling with seven templates is used for Zero-shot CLIP, CLIP-Adapter, and Tip-Adapter.

Table 1 shows the evaluation results. Compared to Tip-Adapter, as a training-free CLIP adaptation method, Proto-Adapter exhibits superior performance across all few-shot settings despite its more compact adapter design. The performance gap widens with an increasing number of training samples, from +0.07 points in the 1-shot setup to 1.86 points in the 16-shot setup. This suggests that Proto-Adapter efficiently aggregates features from multiple training samples into a compact adapter. Remarkably, Proto-Adapter’s performance is on par with that of CLIP-Adapter, which requires fine-tuning, underscoring the proposed method’s potential despite its training-free nature. Furthermore, when fine-tuned with the proposed method, Proto-Adapter-F achieves the best performance among the methods compared for all few-shot settings, except in the 1-shot scenario.

### 3.3. Performance on Diverse Datasets

Table 2 shows the performances of the proposed methods on 11 datasets: ImageNet [10], StanfordCars [19], UCF101 [20], Caltech101 [21], Flowers102 [22], SUN397 [23], DTD [24], EuroSAT [25], FGVCAircraft [26], OxfordPets [27], and Food101 [28]. The adaptation methods for CLIP that do not require training, such as the Proto-Adapter and Tip-Adapter, significantly exceed the average accuracy of CLIP. This improvement is particularly notable in datasets consisting of fine-grained categories, such as FGVCAircraft, DTD, and EuroSAT. These results suggest that these CLIP-adaptation methods effectively utilize the features of a small amount of learning data to efficiently add new domain knowledge to CLIP. Proto-Adapter outperforms Tip-Adapter across all datasets, with the exception of FGVCAircraft. The average accuracy across 11 datasets is 72.69%, which is a significant improvement over Tip-Adapter by +2.37 points. Additionally, Proto-Adapter-F demonstrates performance comparable to that of Tip-Adapter-F, even with a more compact adapter. Notably, on datasets like FGVCAircraft and DTD, which involve specialized recognition tasks such as airplane and texture classification, Proto-Adapter-F shows lower accuracy. This highlights an area for potential improvement in transfer performance on such specialized datasets. Despite these specific cases, the overall results affirm that Proto-Adapter can effectively acquire a lightweight and efficient adapter for various downstream tasks.

### 3.4. Ablation Study

In this section, we conduct several ablation studies for Proto-Adapter. All experiments are conducted on ImageNet, and we adopt the 16-shot setting by default.

**Normalization of Prototypes.** We first examine the impact of normalizing the adapter weights. The evaluation results of applying L2 normalization to different axes of the adapter weights $F_{\mathrm{proto}}\in R^{N\times D}$ are shown in Table 3. Across all application patterns, improvements in performance by L2 normalization are observed. The most substantial improvement is observed when normalization is applied channel-wise (along the first axis) followed by class-wise (along the second axis). The performance gain is more significant with more shots; for instance, a 2.05 point improvement is observed with 16 shots. While we investigate additional normalization strategies, such as standardization, L2 normalization consistently yields the most significant enhancements. These results demonstrate the critical importance of normalizing class prototypes in our proposed approach.

**Angular Margin Penalty m.** We then verify the effectiveness of the angular margin penalty *m* on the model’s performance. A larger margin parameter forces the model to produce more discriminative predictions. Table 4 presents the ablation study results, showing that in all few-shot scenarios, introducing a margin parameter greater than 0 leads to performance enhancements. Notably, a relatively large margin proves to be particularly effective when the amount of training data is limited. This finding implies that metric learning, which aims to increase class separation, is beneficial when working with a limited number of training examples.

**Image Encoders.** Finally, we investigate the impact of CLIP image encoders on our method. Table 5 compares the results of using various sizes of ResNet [29] and Vision Transformer (ViT) [30] as image encoders. Our method consistently outperforms the comparative methods, irrespective of the encoder’s type and size. Notably, when employing ViT/16, the largest model variant, our Proto-Adapter-F surpasses Zero-shot CLIP by 5.5 points. The results demonstrate that Proto-Adapter is effectively adaptable to large-scale pre-trained models for downstream tasks with limited amount of labeled data.

## 4. Discussion

Our proposed Proto-Adapter demonstrates superior recognition performance over other comparative methods in most experimental settings on ImageNet, a generic object dataset. Additionally, its average performance across 11 image classification datasets significantly surpasses that of Tip-Adapter in settings without fine-tuning, and is comparable when fine-tuning is applied. Considering that the Proto-Adapter has a lighter and consistently sized adapter compared to Tip-Adapter, these results are noteworthy.

The key to performance improvement lies in the normalization of prototype vectors. The experimental results suggest that when adapting vision-language models to downstream tasks with limited training data, the construction method of the adapter is more crucial than its size. Moreover, the performance improves when an Additive Angular Margin Penalty is applied during adapter fine-tuning. This supports our hypothesis that introducing distance metric learning in training with limited data can improve the model’s discriminative boundary.

Proto-Adapter also excels in terms of versatility. It can significantly enhance the few-shot performance of the CLIP models regardless of the image encoders. Additionally, the proposed pipeline can potentially be applied to other vision-language models simply by adding a single-layer adapter. Furthermore, Proto-Adapter maintains a consistent and simple architecture regardless of the amount of training data, facilitating its deployment in real-world applications. A model with the proposed adapter can be easily updated when new classes or training data are added. If fine-tuning is unnecessary, a new adapter can be constructed training-free based on Equations (4) and (5). Even with fine-tuning, there is no need for backpropagation to the image–text encoders, and updating only the adapter layer’s weights allows for extremely rapid training (for example, it takes only 8 min on a single NVIDIA GeForce RTX 3090 GPU for the 16-shot setup on ImageNet).

While Proto-Adapter shows superior performance compared to the existing CLIP adaptation methods, there is still room for improvement in performance for specific tasks (e.g., FGVCAircraft and DTD), and there are concerns that outliers may negatively affect model performance. Although these issues are not explicitly addressed in this study to maintain a simple framework, addressing biases and outliers within the training data remains a topic for future work.

## 5. Conclusions

This paper proposes Proto-Adapter, a novel method for adapting CLIP to downstream tasks with limited training data. Our method addresses the issue encountered in the previous study of Tip-Adapter where the size of the adapter is dependent on the quantity of training data by utilizing class-specific prototype vectors as the adapter’s weights. Despite its lightweight and straightforward architecture, our method outperforms Tip-Adapter across various image classification benchmarks. Furthermore, we discover that applying a Additive Angular Margin Penalty, a technique commonly used in metric learning, to fine-tune the adapter significantly enhances the model’s performance. For future research, we envision (1) developing more effective prototype vectors, (2) constructing a universally applicable framework that is effective regardless of training data volume, ranging from few-shot to many-shot scenarios, and (3) expanding our prototype-based adapter to more advanced tasks like semantic segmentation. We hope this study serves as a foundational basis for future research in various directions.

## Figures and Tables

**Table 1:** Performance comparison on ImageNet across different few-shot settings. All comparative methods are based on the pre-trained CLIP with ResNet-50 image encoders. ‘FT’ denotes fine-tuning. Our Proto-Adapter outperforms all other methods in all settings except for the 1-shot scenario.

Method		Shot					
Models	FT	0	1	2	4	8	16
Zero-shot CLIP [13]		60.33	-	-	-	-	-
Tip-Adapter [17]		-	60.70	60.96	60.98	61.45	62.03
**Proto-Adapter**		-	**60.77**	**61.32**	**61.92**	**62.87**	**63.89**
Linear-probe CLIP [13]	✓	-	22.17	31.90	41.20	49.52	56.13
CoOp [16]		-	47.62	50.88	56.22	59.93	62.95
CLIP-Adapter [14]	✓	-	61.20	61.52	61.84	62.68	63.59
Tip-Adapter-F [17]	✓	-	**61.32**	61.69	62.52	64.00	65.51
**Proto-Adapter-F**	✓	-	61.08	**62.05**	**63.05**	**64.49**	**66.17**

**Table 2:** Performance comparison on 11 image classification benchmarks under 16-shot settings. The upper section presents methods without fine-tuning, while the lower section includes methods with fine-tuning.

	ImageNet	Caltech101	OxfordPets	StanfordCars	Flowers102	Food101	FGVCAircraft	SUN397	DTD	EuroSAT	UCF101	Avg
Zero-shot CLIP [13]	60.32	85.92	85.83	55.74	66.02	77.32	17.10	58.52	42.20	37.52	61.35	58.89
Tip-Adapter [17]	62.01	90.43	88.14	66.77	89.89	77.83	**29.76**	66.85	60.93	70.54	70.58	70.32
**Proto-Adapter**	**63.89**	**91.85**	**88.55**	**70.35**	**93.06**	**78.73**	27.21	**69.05**	**64.89**	**75.52**	**76.50**	**72.69**
Tip-Adapter-F [17]	65.51	92.90	89.48	**75.49**	94.19	79.44	**34.92**	71.43	**67.20**	**84.83**	78.54	**75.81**
**Proto-Adapter-F**	**66.17**	**92.90**	**89.56**	75.00	**95.09**	**79.52**	33.00	**71.82**	66.55	83.27	**78.56**	75.59

**Table 3:** Effect of normalizing prototype vectors under different few-shot settings.

Normalization		Shot		
Channel-Wise	Class-Wise	1	4	16
		60.40	60.61	61.84
✓		60.65	60.96	61.75
	✓	60.66	61.34	62.70
✓	✓	**60.77**	**61.92**	**63.89**

**Table 4:** Effect of margin parameter *m* of Additive Angular Margin Penalty. ‘FT’ refers to fine-tuning.

Shot	1	2	4	8	16
w/o FT	60.77	61.32	61.92	62.87	63.89
m=0	60.99	61.66	62.40	63.79	65.56
m=0.1	60.93	61.77	62.72	64.35	65.96
m=0.2	60.86	61.97	62.99	**64.49**	**66.17**
m=0.3	61.02	**62.05**	**63.05**	64.15	65.95
m=0.4	**61.08**	62.01	62.82	63.95	65.54

**Table 5:** Performance results (%) of different methods on various image encoders under the 16-shot setup. Our methods consistently outperform the comparison methods, regardless of the image encoder.

Models	RN50	RN101	ViT/32	ViT/16
Zero-shot CLIP [13]	60.33	62.53	63.80	68.73
Tip-Adapter [17]	62.03	64.78	65.61	70.75
**Proto-Adapter**	**63.89**	**66.81**	**67.07**	**72.25**
CoOp [16]	62.95	66.60	66.85	71.92
CLIP-Adapter [14]	63.59	65.39	66.19	71.13
Tip-Adapter-F [17]	65.51	68.56	68.65	73.69
**Proto-Adapter-F**	**66.17**	**69.12**	**69.27**	**74.23**

## Data Availability

The original contributions presented in the study are included in the article, further inquiries can be directed to the corresponding author.
